# Extracellular Vesicles in Viral Spread and Antiviral Response

**DOI:** 10.3390/v12060623

**Published:** 2020-06-08

**Authors:** Raquel Bello-Morales, Inés Ripa, José Antonio López-Guerrero

**Affiliations:** 1 Departamento de Biología Molecular, Universidad Autónoma de Madrid, Cantoblanco, 28049 Madrid, Spain; inesripa@hotmail.com (I.R.); ja.lopez@uam.es (J.A.L.-G.); 2 Centro de Biología Molecular Severo Ochoa, CSIC-UAM, Cantoblanco, 28049 Madrid, Spain

**Keywords:** extracellular vesicles, exosomes, microvesicles, viral spread, herpesvirus, herpes simplex type 1

## Abstract

Viral spread by both enveloped and non-enveloped viruses may be mediated by extracellular vesicles (EVs), including microvesicles (MVs) and exosomes. These secreted vesicles have been demonstrated to be an efficient mechanism that viruses can use to enter host cells, enhance spread or evade the host immune response. However, the complex interplay between viruses and EVs gives rise to antagonistic biological tasks—to benefit the viruses, enhancing infection and interfering with the immune system or to benefit the host, by mediating anti-viral responses. Exosomes from cells infected with herpes simplex type 1 (HSV-1) may transport viral and host transcripts, proteins and innate immune components. This virus may also use MVs to expand its tropism and evade the host immune response. This review aims to describe the current knowledge about EVs and their participation in viral infection, with a specific focus on the role of exosomes and MVs in herpesvirus infections, particularly that of HSV-1.

## 1. Introduction

Optimization of viral spread is crucial for viruses to maximize their biological success. Once virions have been assembled, matured and released from cells, they must disperse throughout the host and overcome barriers such as selective tropism and the host immune system. In this regard, viruses have evolved strategies to avoid neutralizing antibodies and facilitate dissemination within the host by wrapping themselves in membrane-wrapped clusters [1].

Viral spread by both enveloped and non-enveloped viruses may occur through extracellular vesicles (EVs) [2,3,4,5,6]. EVs have also proven to be an efficient mechanism for viruses to enter host cells or evade the host immune response [7,8,9]. EVs have key biological activities during viral infections, including the transport of viral genomes into target cells and interventions in cell physiology that support infection [10]. Their role as mediators of transplacental and sexually transmitted viral infections has also been proposed [11]. Other advantages ascribed to the use of EVs by viruses include several strategies for dispersing in groups, in so-called “collective infectious units.” One advantage of collective dispersal strategies is an increase in effective multiplicity of infection (MOI) [12]. It has been argued that a high MOI may enhance infectivity by allowing the virus to overcome different infection barriers and may reduce the probability of unsuccessful infections due to stochastic processes. In this regard, propagation as a pool of virions inside EVs can be considered a mode of collective dispersal strategy [12], presenting the concomitant advantages of that *en bloc* viral transmission [13]. Collective infectious units can consist not only of multiple virions inside a vesicle but also of multiple viral genomes within a single virion. The simultaneous delivery of multiple viral genomes to the same cell may have significant consequences for pathogenesis, antiviral resistance and social evolution [14]. In this way, EVs may support genetic cooperativity among viral quasispecies and increase the fitness of the whole viral population [15].

Numerous reports have highlighted the role of EVs in viral infection and their importance in viral entry, spread and immune evasion [2,5,8,9,15,16,17,18]. However, given the complex relationship between viruses and EVs, these vesicles can also trigger anti-viral host responses [6]. In this sense, it is widely accepted that EVs exert critical functions not only in the bolstering but also in the blockage of viral infections, modulating immune responses and serving as a vehicle of intercellular communication between infected and uninfected cells [4,6,19]. Indeed, because of their common biogenesis pathways, EVs and viruses may be considered to be close relatives and it has been argued that a deep understanding of the biology of EVs and the mechanisms by which they impact viral infections is necessary, especially for translation into therapy [20].

Herpes simplex type 1 (HSV-1) may spread by two main pathways—by cell-free virus; or by direct cell-to-cell spread, in which the viral transmission occurs through cell-to-cell contact [21], with cell adhesion proteins and their cytoskeletal connections used for lateral spread [22]. In cell-free virus infections, progeny viral particles must “escape” into the extracellular space and then infect another cell from the outside. Interactions of viral envelope proteins with the cell surface define cell-free viral spread and although it enhances dissemination by allowing diffusing virions to infect distant cells, there are some disadvantages, as free virions can be neutralized by antibodies or subjected to opsonization and phagocytosis [23]. Acquisition of an outer envelope may help shield HSV-1 from neutralizing antibodies while reinforcing viral dissemination [24]. Some strains of HSV-1 can also spread through syncytium formation, which occurs upon fusion of infected cells with neighboring uninfected ones [25].

This review describes current knowledge about the involvement of EVs in viral infections. We specifically focus on recent research on the role of EVs, both exosomes and microvesicles (MVs), during herpesvirus infections, particularly that of HSV-1.

## 2. EVs: Brief Overview

**Figure 1:**
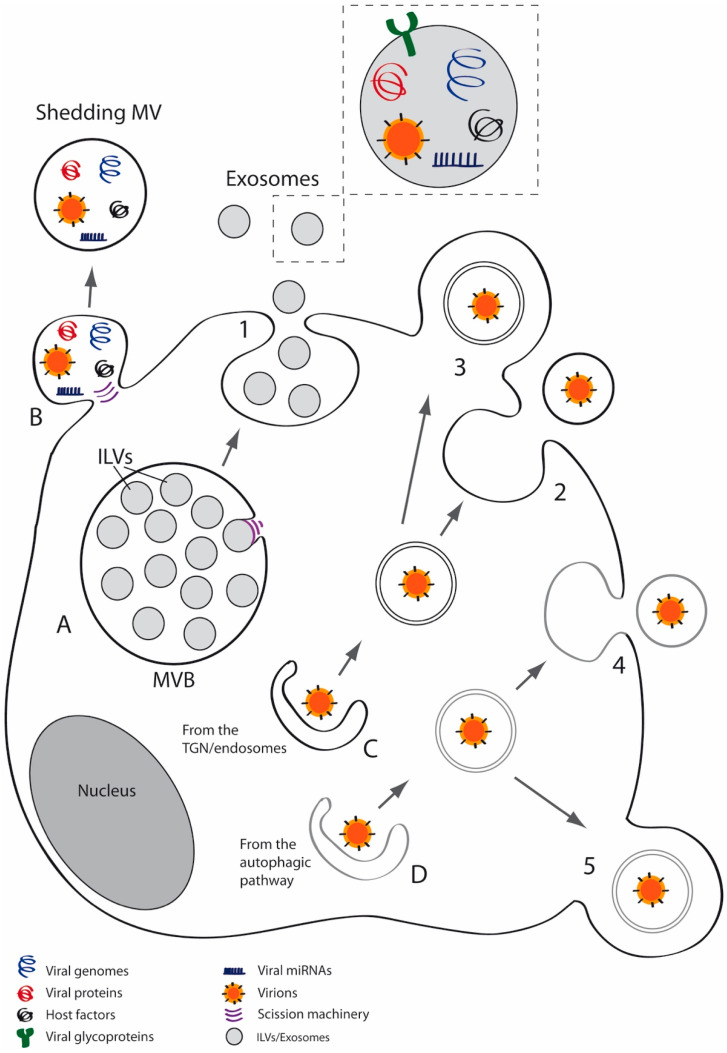
Schematic models of viral spread via extracellular vesicles (EVs). The EV-mediated release of virions, viral components and induced/altered host factors by infected cells, may be performed using different pathways and molecular machineries. Several viral components such as DNAs, RNAs, miRNAs, and/or proteins, may be packaged into EVs. Infected cells may also release EVs containing host factors such as DNAs, RNAs, miRNAs and/ or proteins, induced or altered by the infection. All these components may trigger both pro- or anti-viral effects. A. Viruses may use the multivesicular bodies (MVBs) for viral spread, packaging virions or viral factors into intraluminal vesicles (ILVs) that will be released to the extracellular medium (exosomes) after fusion of the MVB with the plasma membrane (1). B. Viruses may also exit cells enclosed in shedding microvesicles (MVs), which might also contain viral and/or host factors. Other feasible routes for viral spread involve the secretory (C) and the autophagic (D) pathways. C. Several viruses use the TGN or endosomes for envelopment. In the canonical pathway for HSV-1, the viral egress involves the fusion of a double-membrane vesicle with the plasma membrane (2), giving rise to free enveloped virions. Hypothetically, this structure might exit the cell after shedding of the plasma membrane (3), giving rise to a three-membraned EV, which would correspond to an enveloped virion enclosed within a shedding MV. D. Viruses can also be wrapped into vesicles/tubules belonging to the autophagic pathway. These vesicles containing virions could also be released by fusion (4) or by membrane shedding (5). For routes A-D, different molecular machineries, such as the endosomal sorting complexes required for transport (ESCRT) complex, tetraspanin-enriched microdomains (TEMs) and/or lipid rafts, may operate and collaborate for vesicle scission.

Extracellular vesicles (EVs) are membrane vesicles secreted by most cell types which have been isolated from several biological fluids such as blood, urine, cerebrospinal fluid and saliva [26,27,28,29]. Almost all cell types belonging to the three domains of life, Archaea, Bacteria and Eukarya, may secrete EVs [30,31]. Classification and nomenclature of EVs is complex but three major categories of EVs can be broadly established—apoptotic bodies, MVs and exosomes [26,32]. MVs derive from shedding of the plasma membrane [28,33], whereas exosomes are vesicles released to the extracellular space after fusion of multivesicular bodies (MVBs) with the cell membrane [26,32,34] (Figure 1). Exosomes have a typical diameter of 30–100 nm while MVs have a heterogeneous size, ranging from 100 nm to 1 μm in diameter [29,35]. MVs are enriched in lipid rafts and proteins such as flotillin-1 or integrins and expose phosphatidylserine (PS) on the outer plasma membrane leaflet [36,37,38], whereas exosomes are enriched in tetraspanins such as CD9, CD63 and CD81 and endosomal markers including ALIX and TSG101. The relative centrifugal force needed to isolate MVs is frequently between 10,000 and 20,000× *g* [39] and around 100,000× *g* is typically used to pellet exosomes [40,41,42,43].

EVs may be taken up by recipient cells by endocytosis or fusion with the plasma membrane and, given the diversity of EVs, these vesicles can enter cells using different routes. Endocytic pathways are heterogeneous and may include several mechanisms, such as endocytosis that is dependent or independent of clathrin, caveolin-dependent uptake, phagocytosis, macropinocytosis and lipid raft-mediated endocytosis [44]. EVs may be key mediators of several physiological and pathological processes [27,45,46] and are currently associated with cancer [47,48,49,50], infection [3,8,51], inflammation and immune responses [52] and myelination and neuron–glia communication [53,54,55,56,57]. Regarding virus-host interactions, EVs have emerged as a relevant object of attention because of their participation in the intercellular communication processes during viral infections.

## 3. EVs in Viral Infections

### 3.1. Non-Enveloped Viruses

EVs may modulate the infection of diverse viruses and non-enveloped viruses in particular exploit them to exit from cells non-lytically and to avoid and manipulate the immune system [15]. The canonical separation between enveloped and non-enveloped virus has been nuanced by the existence of non-enveloped viruses that, during a major part of their viral cycle, may spread while enclosed in vesicles, thus behaving as “quasi-enveloped” viruses. In this regard, several picornaviruses can be released from their host cells enclosed in EVs. This was first reported in hepatitis A virus (HAV), a hepatovirus which was found enclosed in host-derived membrane vesicles resembling exosomes which protected the virions from antibody-mediated neutralization [58]. The HAV structural protein pX was shown to interact with ALIX to promote the secretion of virions through exosome-like vesicles [59]. These quasi-enveloped viruses were infectious and circulated in the blood of infected humans and their biogenesis was dependent on host ESCRT machinery. Besides HAV, hepatitis E virus (HEV) can be also released through MVBs by the cellular exosomal pathway [60] and may circulate in the blood completely enclosed in membranes during infection, which is just as infectious as their naked counterparts [61]. Unlike enveloped viruses, quasi-enveloped viruses lack viral glycoproteins within the surrounding lipid bilayer but they may display internal proteins (such as VP1pX in the case of quasi-enveloped HEV) that are absent in canonical naked virions. The acquired membrane protects these viruses from neutralizing antibodies while facilitating spread within the host and, like enveloped viruses, they may hijack the host ESCRT machinery to exit infected cells non-cytolytically. In an alternative model, these viruses may also use autophagosome-mediated exit without lysis (AWOL), releasing virions enclosed in LC3-positive vesicles [61]. According to the AWOL model, which was first described for poliovirus release [62], viruses confined in double-membrane structures derived from the autophagic pathway can be released to the extracellular milieu via fusion with the plasma membrane. Another picornavirus, the aphthovirus responsible for foot-and-mouth disease (FMDV) was long ago observed to be released from cells by an exocytic mechanism involving membrane-limited vesicles [63]. More recently, a mechanism of exosome-mediated transmission of FMDV has been described in vivo and in vitro [64] and has also been considered as a potential immune evasion mechanism.

Cells infected by Coxsackie B virus (CBV) have demonstrated to release EVs. Thus, infected neural progenitor and stem cells (NPSCs) and C2C12 myoblast cells induced the release of abundant MVs containing viral proteins and infectious virus, a process that meant a novel route for virus dissemination [65]. Cells infected with CBV trigger fragmentation of mitochondria through a precursor of autophagic mitochondrial elimination (mitophagy) and virions may be released within MVs derived from these mitophagosomes [66]. The acquisition of this “cloak” may help the virus to evade the immune system, allowing an efficient non-lytic viral spread. CBV can also enhance replication efficiency by packaging microRNAs (miRNAs) into EVs from infected cells [67].

Enterovirus 71 (EV71) can be transported non-lytically between cells during early viral infection and exosomes containing this virus have been shown to establish a productive infection in human neuroblastoma cells [68]. EV71 virions were also found inside exosomes from infected human rhabdomyosarcoma cells and also have been detected in human samples [69]. Upon infection, another picornavirus, encephalomyocarditis virus (EMCV), triggers the release of multiple EV subpopulations that differ in their physical properties, composition and function [70].

Choroid plexus epithelial cells infected with the human polyomavirus 2 (JC virus) may produce EVs containing virions [71]. Those vesicles expressed exosomal markers such as CD9 and TSG101 and entered glial cells by macropinocytosis and clathrin-dependent endocytosis. The presence of virions in EVs may constitute a main pathway for its spread, since oligodendrocytes and astrocytes, the major targets of JC virus in the central nervous system (CNS), do not express the viral attachment receptors needed for direct viral fusion. EVs may also mediate the interaction between the human papilloma virus (HPV) and human immunodeficiency virus (HIV)-1. In this way, exosomes secreted from cells infected with HPV may increase HIV-1 replication in U1 cells via an oxidative stress pathway and treatment with antioxidants reduced EV-mediated enhancement of HIV-1 replication [72].

Gastroenteric non-enveloped pathogens such as noroviruses and rotaviruses may also spread enclosed in EVs, transferring a higher infectious dose to the next host cell and contributing to enhanced fecal-oral propagation [73]. Bluetongue virus (BTV) is a reovirus that may also be released from cells by a budding process. The majority of virions are released by cell lysis in mammalian cells but in insect cells, the release of BTV is nonlytic. Expression of NS3, a non-structural viral protein, in invertebrate cells infected with this arbovirus has been reported to correlate with nonlytic virus release. NS3 upholds virus release by recruiting ESCRT-I protein TSG101 [74] and it may act like the membrane protein of enveloped viruses, being responsible for intracellular trafficking and budding of virus particles [75]. The integrity of MVBs is also important for BTV assembly [76], as it occurs for other enveloped viruses.

### 3.2. Enveloped Viruses

Several enveloped viruses exploit EVs as a vehicle for enhancing their propagation. For example, EVs play a relevant role in hepatitis C virus (HCV) spread, as virions contained in exosomes can be transported to hepatocyte-like cells, establishing a productive infection [77]. Exosomes found in the serum of HCV infected patients can also mediate HCV transmission to hepatocytes [78].

Exosomes released by influenza virus-infected cells may modulate immune response through its transfer of different cargoes such as proteins, mRNAs or miRNAs. Upon infection, the expression of host miRNAs is altered and those miRNAs can regulate viral genes and both stimulate or suppress cell apoptosis and innate immune responses during infection. Therefore, secretion of exosomes and dysregulation of miRNAs are associated with immune regulation and pathogenicity during infection with this virus [79]. In mouse model, infection with influenza increases the levels of miRNAs in exosomes from bronchoalveolar lavage fluid. Moreover, serum exosomes from mice infected with influenza showed high levels of miR-483-3p (one of those miRNAs). The addition of those exosomes to MS1 murine cell line and their following uptake by those cells, potentiated the expression of proinflammatory cytokines in that vascular endothelial cell line [80]. A Y5 RNA-derived small RNA, Hsa-miR-1975, was secreted in exosomes and transferred to neighboring cells to induce interferon expression, which highlights the role of this Y-class small RNA in host’s defense against influenza as an antiviral mechanism based in exosomal traffic [81].

EVs may also modulate infections carried out by flaviviruses such as Dengue virus (DENV). Upon DENV infection, immune cells increase secretion of pro-inflammatory factors into the bloodstream, causing hyperpermeability. It has been hypothesized that exosomes and/or MVs might be hijacked to benefit viral spread and pathogenesis, perhaps triggering hyperpermeability and plasma leakage [82]. The versatility of the biological role of EVs is exemplified by virus-vector-host interactions that limit infection in mosquito cells. Host EVs may enter mosquito cells, inhibiting their infection through the restriction of virus-endosomal membrane fusion [83]. In addition, exosomes released from DENV-infected macrophages and added to the human EA.hy926 endothelial cells induced physiological changes in that cell line, leading to a protective effect during the early stages of infection that may help to maintain endothelial integrity [84]. Exosomes from C6/36 mosquito cells infected with Zika virus (ZIKV) may also modify host cells responses and contribute to the pathogenesis of ZIKV infection in humans. These exosomes induced a pro-inflammatory state and participated in endothelial vascular cell damage by inducing coagulation, inflammation and endothelial permeability [85].

EVs may also modulate retroviral pathogenesis [86,87]; HIV and simian immunodeficiency viruses modulate vesicle secretion through the Nef protein, which is secreted in exosomes and modifies intracellular trafficking pathways, enhancing viral infectivity and regulating host gene expression and immune responses [88,89,90,91]. In addition, TNFα release by cells upon incorporation of exosomes secreted by infected cells may reactivate latent HIV-1 [92]. The “Trojan exosome” hypothesis states that retroviruses use cellular exosome biogenesis pathways to form infectious particles and the exosome uptake pathway for receptor-independent, Env-independent routes of infection [93]. In other words, retroviruses coopt the cellular machinery for exosomal release; the strong concordance between the host exosome protein profile and that of HIV-1 supported this hypothesis [94], although other authors have questioned the existence of this type of specialized and shared release pathway for HIV-1 and exosomes [95]. Later reports supported the Trojan exosome hypothesis [96,97], proposing a model in which dendritic cells (DCs) internalize retroviruses by endocytosis and subsequently infect interacting CD4+ T cells, a mechanism known as trans-infection that may coexist with direct infection to a different extent depending on the maturation state of the DCs [98].

EVs are involved in the pathogenesis of other retroviruses, too. For instance, EVs produced by cells infected with human T-cell lymphotropic virus-1 (HTLV-1) may transport viral proteins and RNA, causing adverse effects on recipient uninfected cells and increasing viral spread via the upregulation of cell-to-cell contacts [99].

Exosomes may be key mediators of immune responses in patients with respiratory viral infections. Exosomes were detected in serum samples collected from lung transplant recipients with symptomatic respiratory infections caused by coronavirus, respiratory syncytial virus and the non-enveloped rhinovirus, as well as from non-symptomatic stable recipients. Those exosomes enclosed significantly higher levels of lung self-antigens, 20S proteasome and viral antigens when compared with controls. When mice were immunized with those exosomes obtained from transplant recipients with respiratory viral infections, they developed immune responses to self-antigens, small airway occlusion, fibrosis and cellular infiltration [100].

## 4. EVs in Herpesvirus Infections

### 4.1. Alphaherpesviruses

Alpha-, beta- and gammaherpesviruses may exploit EVs to enhance viral spread or may trigger EV-mediated host immune responses [101,102]. The relationship between EVs and HSV-1 is the most heavily investigated among the subfamily Alphaherpesvirinae. HSV-1 is a double-stranded DNA enveloped virus. It is a highly prevalent neurotropic human pathogen [103] that can establish latency in neurons [104]. Primary infection occurs in epithelial cells and then the virus spreads to neurons of the trigeminal ganglia, travelling in a retrograde direction toward the neuron cell bodies, where the virus establishes latent infections [105]. To establish and maintain latency, HSV-1 expresses miRNAs that can downregulate key viral immediate early proteins [106]. Herpesviruses, in general, use such miRNAs to induce and maintain latency [107].

Around 90% of people are seropositive for HSV-1 −which indicates a past exposure to the virus− and have latent HSV-1 genomes in the trigeminal ganglia. Although early studies discovered latent HSV-1 and HSV-2 in the trigeminal and sacral ganglia, respectively, both viruses may also spread to spinal ganglia [108]. Latent HSV-1 can reactivate periodically, either spontaneously or following different triggering events such as ultraviolet light exposure, immunosuppression, fever or x-ray irradiation [109]. Under certain circumstances, HSV-1 may cause severe pathologies such as keratoconjunctivitis or encephalitis [110], which is the major cause of sporadic fatal encephalitis worldwide. HSV-1 is also an increasing cause of genital herpes [111].

HSV-1 may infect many different hosts and cell types [112] using different receptors and different pathways—plasma membrane fusion at neutral pH; or endocytosis that can be dependent or independent of low pH [113,114,115]. In many cultured cell lines, such as HEp-2 and Vero, HSV-1 enters cells by a pH-neutral fusion with the cell membrane. However, it enters HeLa and CHO-K1 cells by endocytosis and subsequent exposure to a low pH [116]. Heparan sulfate glycosaminoglycans operate as attachment receptors for the viral glycoprotein gC [117]. Although this glycoprotein is not essential for viral entry, its absence decreases infectivity, due to the reduction of efficiency of viral binding to cells [116]. In the absence of gC, gB can mediate binding to heparan sulfate [118]. Regardless of the pathway, gD, fusion effector gB and fusion modulator complex gH/gL are essential for HSV-1 entry [119]. Capsid assembly and DNA packaging occur in the nucleus, whereas primary envelopment and de-envelopment take place at the nuclear envelope. Acquisition of tegument and secondary envelopment occurs in the cytoplasm, via budding into vesicles derived from the trans-Golgi network (TGN coated with viral glycoproteins and additional tegument proteins [120]. As with other viruses, MVBs may affect significantly the HSV-1 envelopment and egress, since MVBs modified by the virus constitute a site for envelopment of this virus [121,122].

Finally, viral particles may be released by exocytosis at the plasma membrane and/or transmitted by cell-to-cell at cell junctions [120]. In human tissues, the major mode of HSV-1 transmission is cell-to-cell spread, that is, the direct passage of progeny virus from an infected cell to an adjacent one; this mechanism may serve as an immune evasion strategy, since it protects the virus from immune surveillance [123]. The main entry receptors for gD glycoprotein are HVEM [9], nectin-1 [10] and 3-O-sulfated heparan sulfate [11]. Paired immunoglobulin-like type 2 receptor (PILR) alpha [124] and myelin-associated glycoprotein (MAG) [125] are receptors for gB. Many details about the process of viral dissemination are not completely understood yet and, therefore, clarifying the mechanisms of viral spread and subsequent entry into other cells is still required to fully understand the viral cycle. In this respect, deepen into the role of EVs during HSV-1 infection will shed light on this complex process.

The production of secreted vesicles by cells infected with HSV-1 has been known for a long time and is currently well described. The first to be discovered were the light particles (L-particles), which are similar to virions in appearance but lack the viral nucleocapsid and genome and therefore are not infectious [126,127]. L-particles are secreted after infection with all alphaherpesviruses tested to date, both in human and animal cells [128]. Although L-particles are not infectious, they may deliver viral proteins and cellular factors needed for virus replication and immune evasion such as ICP0, ICP4 and gB [129,130], thus facilitating HSV-1 infection. L-particles and virions show common features—vhs (virión host shutoff, a protein located in the tegument that selectively degrades mRNA early in infection) and α-TIF (a tegument protein that induces immediate early genes by interacting with two cellular proteins) exert the same function in both particles and both share similar assembly and egress pathways, suggesting that viral glycoproteins and tegument are sufficient to induce secondary envelopment [120,127]. There are another type of particles, pre-viral DNA replication enveloped particles (PREPs), which share morphological features with L-particles but differ in relative protein composition. For instance, gC and gD are reduced or absent in PREPs whereas VP22 or gE are increased [131].

Transfer of functional viral proteins to uninfected cells via L-particles may suggest a viral immune escape strategy. Incubation of mature dendritic cells with L particles reduced CD83 (a molecule with costimulatory properties) expression on uninfected bystander dendritic cells, showing that functional viral proteins may be transmitted via L particles from infected to uninfected bystander cells, thereby inducing CD83 downregulation [130]. Similarly, HSV-1 may also exploit the EV pathway to evade the immune system. In this regard, it has been demonstrated that this virus may manipulate the MHC class II processing pathway by modifying the endosomal sorting and trafficking of HLA-DR, hijacking these molecules from their typical cell membrane function and instead diverting them into the exosomal pathway [132]. Likewise, work in our laboratory showed that infection of Chinese hamster ovary (CHO) cells with virions enclosed in MVs was not completely neutralized by anti-HSV-1 antibodies, suggesting that these EVs may be shielding the viral particles from the immune system [133].

However, immune modulation by EVs is complex and multifaceted. Recent studies have described the release of EVs, ranging between 50 and 110 nm, from cells infected with HSV-1. Those vesicles carried viral and host transcripts −mRNAs, miRNAs and non-coding RNAs− and proteins, including the tetraspanins CD9, CD63 and CD81 and innate immune components such as stimulator of IFN genes (STING) [134]. STING activates transcription of type I IFNs, which induce an antiviral response. The use of exosomes to export STING to uninfected cells has been explained as a way to control dissemination of the virus, in such a way that HSV-1 might limit the spread of infection from cell-to-cell to control its virulence and facilitate the dissemination between individuals [129,134,135].

It has been reported that Rab27a, a small GTPase implicated in secretion of exosomes [136], may influence the assembly of viruses such as HIV-1 [137] and HCMV [138]. Likewise, studies carried out by our group showed that this GTPase plays a relevant role in HSV-1 infection of oligodendrocytes [139]. Our results showed a significant reduction in plaque size and viral production in Rab27a-silenced cells infected with HSV-1, suggesting that Rab27a depletion may affect viral egress. More recent studies have revealed the participation of MVs in HSV-1 spread. Our study [133] described the features of MVs released by different cell lines infected with HSV-1 and their participation in the infectious cycle, suggesting that MVs released by cells infected by HSV-1 contained viral particle and were endocytosed by naïve cells, leading to productive infection. These findings suggested that HSV-1 spread might use MVs to expand its tropism and evade the immune response [133,140]. The exact process of HSV-1 targeting to MVs is not known yet but our results suggest that autophagy may be involved in that process, since MVs isolated from infected cells were positive for LC3-II, an autophagy marker. Hence, those results point to a role for the autophagic pathway in the MVs-mediated HSV-1 spread [140].

Though HSV-1 is the best-studied alphaherpesvirus, other species belonging to this family have also been involved in secretion and/or modulation of EVs. Bovine herpesvirus-1 (BoHV-1) and pseudorabies virus (PRV) may also localize gB to EVs. This glycoprotein co-localized with CD63 and MHC II in late endosomes [141]. On the other hand, exosomes secreted by lymphocytes after infection with varicella-zoster virus (VZV) were found to contain selectively concentrated STING [142], suggesting that this virus may modulate the immune system via exosomes.

### 4.2. Betaherpesviruses

The exosome secretion pathway plays a significant role in the life cycle of human herpesvirus 6 (HHV-6), a betaherpesvirus that can modify the molecular transport machinery in infected cells. HHV-6 virions are released along with intraluminal vesicles via the exosomal pathway, by fusion of the limiting membrane of MVBs −in which virus particles and exosomes are enclosed− with the plasma membrane [143]. In addition, T cells infected with HHV-6 showed reduced surface and intracellular expression of MHC class I molecules but these molecules were redistributed to TGN- or post-TGN-derived vesicles and then incorporated into virions and exosomes. The reduction in the total expression of MHC class I in HHV-6-infected cells suggests that a fraction of these molecules may be transported to lysosomes and degraded through the same route that is used to transport particles to MVBs [144]. This mechanism may be important for the virus to evade immune surveillance [101].

A relevant role for MVBs in the release of human cytomegalovirus (HCMV) has been also reported. The association of HCMV proteins with MVBs has been demonstrated [145] and participation of MVBs in the final envelopment of HCMV has also been reported [146]. The presence of gB and gH (two envelope glycoproteins that are essential for HCMV infectivity) in exosomes secreted by HCMV-infected cells has also been recently observed [147]. The exosomes secreted by HCMV-infected cells might exert a relevant effect on the immune system. Thus, endothelial cells infected with HCMV generated viral antigens associated to ALIX-, TSG101- and CD63-positive exosomes that indirectly activated CD4+ T cells. This suggests that circulating exosomes from the HCMV-infected vascular endothelium might be a source of HCMV antigens in infected individuals, hypothetically contributing to the establishment of a HCMV-specific memory T cell population [148].

### 4.3. Gammaherpesviruses

The human gammaherpesviruses Epstein-Barr virus (EBV) and Kaposi’s sarcoma-associated herpesvirus (KSHV) have also been demonstrated to modulate the tumor microenvironment through exosomes. Thus, these oncogenic viruses may modify the protein content of exosomes to modulate the tumor microenvironment, enhance viral efficiency and promote tumorigenesis [149,150,151].

To contribute to the development and progression of malignancy, oncogenic viruses such as EBV promote a pro-tumoral environment, often by altering vesicle content and secretion. EBV-infected cells secrete exosomes carrying viral factors that can be internalized by recipient cells. For instance, exosomes released from latently-EBV-infected nasopharyngeal carcinoma cells (NPC) contained the latent membrane protein-1 (LMP1), signal transduction molecules and virus-encoded miRNAs [151]. LMP1 is the principal oncogene of EBV, being expressed in most EBV-related cancers and is critical for B cell immortalization and cellular transformation [152]. This viral oncoprotein, expressed by cells latently infected with EBV, critically contributes to pathogenesis by deregulation of cellular signal transduction pathways [153]. It has been recently shown that LMP1 promotes EVs secretion and may enhance cancer progression and metastasis by up-regulating syndecan-2 (SDC2) and synaptotagmin-like-4 (SYTL4) through nuclear factor (NF)-κB signaling [154].

LMP1 was initially detected by immunoelectron microscopy in budding plasma membrane protrusions compatible with MVs, although this oncoprotein was also found in the pellet obtained from the centrifugation of conditioned medium at 70,000× *g* [150]. A simultaneous report found that exosomes containing LMP1 inhibited the proliferation of peripheral blood mononuclear cells, suggesting that this viral oncogene may be involved in immune regulation helping the infected tumor cells to escape the immune system [155]. Inhibition of immune responses may not only promote viral spread but also its oncogenic transformation [17]. In addition, LMP1 associates with CD63 in endosomes and its secretion in exosomes reduces NF-κB activation, a relevant fact given that constitutive activation of NF-κB by LMP1 stimulates the proliferation of EBV-infected cells to establish viral persistence [156]. Therefore, since LMP1-modified exosomes enhance the growth, migration and invasion of malignant cells and thus enhance progression of EBV-associated tumors, the localization of LMP1 in exosomes is critical for tumor progression. The role of CD63 in this process is also crucial, since CD63 knockout resulted in a reduction of LMP1-induced particle secretion and LMP1 packaging impairment [157]. EBV-encoded LMP1 may also contribute to upregulation of intercellular adhesion molecule 1 (ICAM-1) [158].

On the other hand, exosomes can also transfer functional miRNAs from EBV-infected cells to subcellular sites of gene repression in uninfected recipient cells [159] and exosomes carrying miRNAs have been detected in blood samples from nasopharyngeal carcinoma patients [160]. The main functions of EBV-encoded miRNAs might be related to immune evasion, inhibition of apoptosis and cell transformation and proliferation, whereas the cellular miRNAs modulate their own biogenesis and latent/lytic infection [161]. A high expression of viral miRNAs encoded by human herpesviruses in diseased human tooth pulps has been recently demonstrated [162]. Examination of their influence on cellular miRNA of primary human oral keratinocytes showed high levels of viral miRNAs in exosomes derived from viral miRNA-transfected oral keratinocytes. In addition, those exosomes released their contents into macrophages, altering expression of their endogenous miRNAs, suggesting that herpesvirus-encoded miRNAs produced during oral infection might impact host defenses and exacerbate pathogenesis in oral inflammatory diseases generally considered to be of bacterial origin [162]. Herpesvirus-encoded miRNAs within exosomes have also been implicated in other diseases such as lichen planus, a chronic inflammatory disease with unclear etiology that has been associated with secretion of exosomes by cells infected with HCMV and other herpesviruses [163,164]. In addition, the miR-200 family of miRNAs acts as a cellular switch, regulating the shift from latency to the lytic cycle. In this context, it has been shown that miRNA-200 are packed into exosomes which create an epithelial microenvironment that promotes the EBV lytic cycle [165].

Exosomes may coat bystander lymphocytes with IL-35, an immunosuppressive cytokine composed of EBV-induced protein 3 (Ebi3) and IL-12α chain (p35) subunits. This EV-associated cytokine promotes infection tolerance in two ways—first, by inducing IL-35 production in non-Treg (regulatory T) cells; and second, by causing an immunosuppressive phenotype in EV-acquiring T and B cells, leading to secondary suppression of immune responses [166]. Another recent study has described a process due to a paracrine loop in which EBV M81 strain-infected B cells secreted exosomes, containing EBER2 RNA, that were endocytosed by neighboring cells. This RNA increased CXCL8 expression, a chemokine that enhanced spontaneous lytic replication levels in M81-infected B cells [167].

KSHV is the causative agent of Kaposi’s sarcoma, the most frequent cancer in untreated HIV patients suffering from acquired immunodeficiency syndrome (AIDS). Early studies showed that KSHV greatly alters the protein content of exosomes and that exosomes secreted from B cells infected with KSHV affect cellular metabolism and likely modulate cell death and survival [149]. It is widely accepted that the exosomal pathway is exploited by KSHV for viral spread and oncogenesis [168]. Exosomes obtained from patient primary effusion lymphoma pleural fluid produced an earlier and enhanced migration of endothelial cells, giving these patient-derived exosomes a functional biological role in cell migration Therefore, the exosomes derived from KSHV-associated malignancies were functional and in addition contained a distinct subset of miRNAs [169]. Later studies have demonstrated that exosomes isolated from the saliva of HIV patients and secreted by HIV-infected T-cell lines stimulated KSHV infectivity in epithelial cells, revealing that HIV-associated exosomes are a risk factor for KSHV infection in HIV-infected patients [170].

## 5. The Role of Lipid Rafts and the MAL Proteolipid

Myelin and lymphocyte protein (MAL) [171] is nonglycosylated integral membrane protein with four hydrophobic domains located in detergent-insoluble membrane fractions enriched in condensed membranes [172]. This proteolipid is the prototypical member of the MAL family, which reside in detergent-insoluble membranes enriched in cholesterol and glycolipids [173]. MAL is expressed in several cell types including epithelial and endothelial cells, hepatocytes and T cells that are found in several tissues [174]. In the nervous system, MAL is predominantly localized in compact myelin formed by oligodendrocytes and Schwann cells, playing an essential role in the stability of myelin [175] and regulating the distribution of PLP, the main myelin protein, into different microdomains [176]. This proteolipid plays a critical role in apical transport in epithelial cells [177,178], whereas in T lymphocytes, MAL is located at the immunological synapse and significantly affects exosome secretion [179,180].

The ESCRT machinery is typically involved in the budding and release of endosomal vesicles into MVBs. However many viruses, such as HIV or Ebola, utilize this machinery for budding and release. ESCRT proteins, which are localized in the neck region of membrane buds, mediate the membrane scission for viral release. Enveloped viruses acquire their lipid bilayers by budding through host membranes and many of them hijack the cellular ESCRT machinery to exit from the cells [181]. Some non-enveloped viruses such as BTV and HAV can also recruit the ESCRT pathway for release. However, the budding of other viruses, such as influenza, is ESCRT-independent. Lipid rafts substantially contribute to viral entry and assembly of enveloped and non-enveloped viruses [182] and they also contribute to the budding of several enveloped viruses via accumulation of viral components at the budding site, thus facilitating their interaction [183]. Some ESCRT proteins were identified in lipid rafts and it is probable that these proteins could associate to these lipid microdomains to promote membrane budding. Thus, the ESCRT proteins could bind to pre-existing rafts or induce lipid domain formation. Lipid rafts are a platform for assembly of many viruses and, therefore, the enrichment of ESCRT proteins in lipid rafts would facilitate their recruitment to assembling virions [184]. The role of TEMs in viral budding and release has also been demonstrated for several viruses [185].

The involvement of MAL in HSV-1 infection has been recently reported [186]. HSV-1 virions were transported in association with MAL-positive structures in oligodendrocytes to reach the end of cellular processes, which contact uninfected cells. In addition, as functional studies showed, the depletion of MAL led to a significant decrease in infection, with a drastic reduction in the number of lytic plaques in MAL-silenced cells. In this context, MAL proteolipid might be involved in viral spread via two main pathways—first, through its role in raft-mediated direct transport; and second, through its function on exosome secretion. In the absence of MAL, the formation and traffic of MVBs may be greatly impaired, because tetraspanin-enriched microdomains are not incorporated efficiently into intraluminal vesicles of MVBs. MAL accumulates on the limiting membrane of MVBs, triggering the rerouting of these aberrant MVBs to lysosomes for degradation. Therefore, MAL might exert a multifaceted role delivering virions either to cell-to-cell contacts or the plasma membrane or, otherwise allow their incorporation into MVBs. The detailed participation of the MAL proteolipid in viral spread is worth future investigation.

## 6. Conclusions

Both enveloped and non-enveloped viruses may exploit EVs to enhance their viral cycle but may also be the object of antiviral EV-mediated responses. The majority of viral families contain species that may hijack the EV-mediated endocytic machinery to enter cells and/or use secreted EVs for viral release. In addition, several viruses may interfere with the host immune system. In most cases, viruses use exosome secretion pathways, although viruses such as CBV, EBV or HSV-1 may use shedding MVs for viral spread and immune escape.

Alpha-, beta and gammaherpesviruses can use EVs to enhance viral spread or to modulate the immune response. Regarding HSV-1, exosomes secreted by infected cells may transport viral and host transcripts, proteins and innate immune components and this virus may use shedding MVs to expand its tropism and to evade the host immune response. A deep understanding of EVs and their involvement in host-viral interactions is essential for future use in diagnostics and anti-viral therapy.
